# Endoscopic treatment for complicated gastric antral-embedded foreign bodies: reflection and retrospection

**DOI:** 10.1093/gastro/goaf026

**Published:** 2025-04-07

**Authors:** Jia Yu, Pingting Gao, Shengli Lin, Quanlin Li, Yunshi Zhong, Liqing Yao, Lili Ma, Pinghong Zhou

**Affiliations:** Endoscopy Center and Endoscopy Research Institute, Zhongshan Hospital, Fudan University, Shanghai, P. R. China; Shanghai Collaborative Innovation Center of Endoscopy, Zhongshan Hospital, Fudan University, Shanghai, P. R. China; Endoscopy Center and Endoscopy Research Institute, Zhongshan Hospital, Fudan University, Shanghai, P. R. China; Shanghai Collaborative Innovation Center of Endoscopy, Zhongshan Hospital, Fudan University, Shanghai, P. R. China; Endoscopy Center and Endoscopy Research Institute, Zhongshan Hospital, Fudan University, Shanghai, P. R. China; Shanghai Collaborative Innovation Center of Endoscopy, Zhongshan Hospital, Fudan University, Shanghai, P. R. China; Endoscopy Center and Endoscopy Research Institute, Zhongshan Hospital, Fudan University, Shanghai, P. R. China; Shanghai Collaborative Innovation Center of Endoscopy, Zhongshan Hospital, Fudan University, Shanghai, P. R. China; Endoscopy Center and Endoscopy Research Institute, Zhongshan Hospital, Fudan University, Shanghai, P. R. China; Shanghai Collaborative Innovation Center of Endoscopy, Zhongshan Hospital, Fudan University, Shanghai, P. R. China; Endoscopy Center and Endoscopy Research Institute, Zhongshan Hospital, Fudan University, Shanghai, P. R. China; Shanghai Collaborative Innovation Center of Endoscopy, Zhongshan Hospital, Fudan University, Shanghai, P. R. China; Endoscopy Center and Endoscopy Research Institute, Zhongshan Hospital, Fudan University, Shanghai, P. R. China; Shanghai Collaborative Innovation Center of Endoscopy, Zhongshan Hospital, Fudan University, Shanghai, P. R. China; Endoscopy Center and Endoscopy Research Institute, Zhongshan Hospital, Fudan University, Shanghai, P. R. China; Shanghai Collaborative Innovation Center of Endoscopy, Zhongshan Hospital, Fudan University, Shanghai, P. R. China

## Introduction

A fully antral-embedded foreign body (FB) often presents with nonspecific symptoms and misleading endoscopic findings, complicating diagnosis and endoscopic removal [1]. Here, we present a series of cases detailing endoscopic extraction of gastric antral-embedded FBs, encapsulating insights from our center’s experience.

## Patient characteristics

Patients with gastric antral-embedded FBs were prospectively enrolled at Zhongshan Hospital, Fudan University (Shanghai, P. R. China) from January 2022 to January 2024. Inclusion criteria were as follows: (i) clinically stable patients without signs of sepsis and (ii) FBs embedded in the gastric antrum without significant contamination. Preoperative computed tomography (CT) scans were used to assess FB morphology, anatomical positioning, and spatial relationships with adjacent vital structures, ruling out macrovascular injury and gastrointestinal perforation. The study was approved by the Ethics Committee of Zhongshan Hospital (B2021-558) and conducted in accordance with the Declaration of Helsinki.

Patient characteristics are detailed in Table 1. Six patients (2 females, 4 males; mean age 56.5 ± 5.0 years) underwent endoscopic removal by one endoscopist (P.Z.). No patient had pre-existing conditions, such as chronic atrophic gastritis, with a median ingestion time of 1 month (range 0.2–24 months). Abdominal pain, with or without distension, was the main symptom in five patients, while one patient reported acid reflux and abdominal pain lasting nearly a week. CT scans revealed strip-like high-density shadows in the antrum, with vague surrounding fat spaces. Endoscopic removal was performed due to localized infection, as the patient remained clinically stable without peritonitis.

**Table 1.** Demographics, clinical characteristics, procedure details, and postoperative management of all cases in this study

Case	Age, year	Gender	FB type	Length,cm	Duration of FB ingestion	Symptoms	Preoperative CT image	Procedure duration, min	Nasogastric tube insertion	Length of hospital stay, days
1	53	M	Fish bone	0.8	5 days	Abdominal distension, abdominal pain	8-mm antrum FB deep into the muscularis propria	25	−	5
2	55	F	Fish bone	4	1 week	Acid reflux, abdominal pain	Antrum FB partly embedded in the stomach wall	40	−	2
3	51	M	−^a^	−^a^	1 month	Abdominal pain	Antrum FB	180	+	4
4	55	M	Hem-o-lok clip	1	2 years	Abdominal pain	–	30	+	3
5	64	F	Metallic wire	1.2	26 days	Asymptomatic	12-mm FB in the distal gastric antrum	30	−	4
6	61	M	Fish bone	3	1 month	Abdominal distension, abdominal pain	Antrum FB with peripheral effusion	60	+	3

F = female, M = male, FB = foreign body, CT = computed tomography.

a No foreign body was found during surgery in this patient.

Endoscopic removal succeeded in five out of six patients, with a mean procedure duration of 60.8 ± 59.7 min (range 25–180 min). The FBs comprised three fish bones, one metal wire, and one Hem-o-lok clip, with a mean length of 2.0 ± 1.4 cm (range 0.8–3.0 cm). Nasogastric tubes were placed in three patients due to incomplete closure of high-tension gastric wall defects. All defects were closed using endo-clips. The mean hospital stay was 3.5 ± 1.0 days (range 2–5 days), with no major adverse events, including bleeding, sepsis, gastric perforation, or peritonitis reported during the 3-month follow-up.

## Technical details

**Figure 1.**
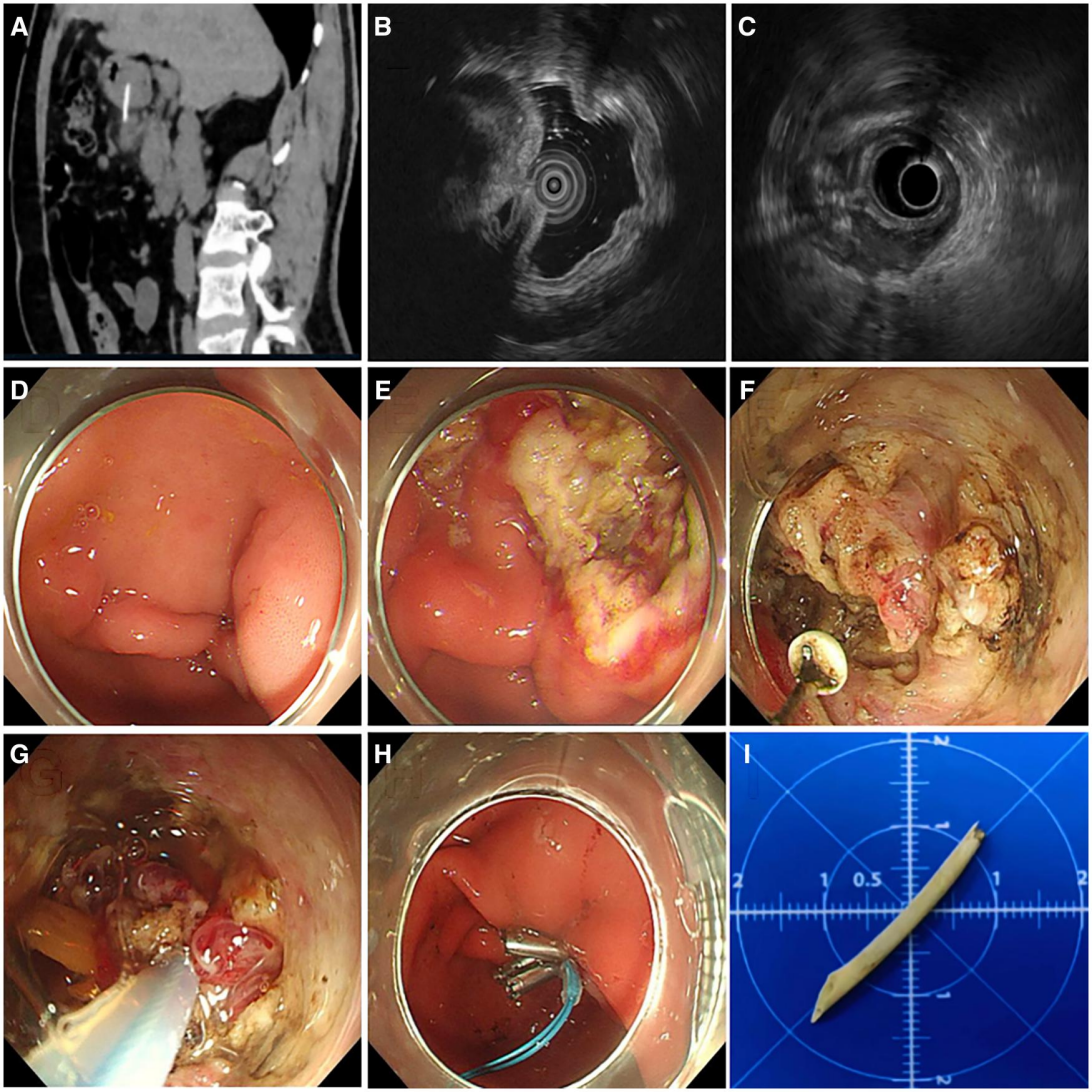
Endoscopic removal of gastric antral-embedded foreign body (FB) in one patient with a 1-month history of abdominal distension and pain. (**A**–**C**) Localization of the FB: pre-operative visualization of the FB under computed tomography (A) and intraoperative 20 MHz (B) and 6 MHz (C) endoscopic ultrasonography. (**D**) Mass with umbilication under endoscopy. (**E**) Submucosal injection and overlying mucosal flap removal. (**F**) Granulation tissue dissection, myotomy, and FB exposure. (**G**) FB extraction. (**H**) Hemostasis and closure of the gastric wall wound. (**I**) The FB was a 3-cm-long linear fish bone.

The procedure was performed in the endoscopy suite by one endoscopist using a standard gastroscope with a transparent hood attached. Other accessories included a hook knife, insulated-tip knife, grasping forceps, hemostatic forceps, and a snare. The procedure involved five main steps (Figure 1 and Supplementary Video 1):

- Step 1: FB localization. Preoperative CT imaging helped identify the embedded FB and related complications. If the CT scan was negative, endoscopic ultrasonography (EUS) was performed preoperatively for FB. Intraoperative EUS was vital for assessing penetration depth and surrounding tissues to determine the optimal incision site. The choice of EUS probe (small or standard) was based on the FB size.
- Step 2: Submucosal injection and overlying mucosal flap removal. A straight distal transparent hood was attached to the endoscope’s tip. Normal saline was injected into the submucosal layer, followed by an incision using a hook knife. The mucosal flap was completely removed with a snare, exposing granulation tissue or the FB tip.
- Step 3: Granulation tissue dissection, myotomy, and FB exposure. Depending on the FB shape, snares or forceps were utilized repeatedly to attempt to grasp the suspicious tissue and ascertain the location of FB. Under EUS guidance, a hook knife with or without insulated-tip knife assisted was employed to dissect surrounding granulation tissue and/or muscle fibers. Grasping forceps and/or a snare were used to help locate FB until the embedded FB was fully exposed.
- Step 4: FB extraction. The exposed FB tip was grasped with grasping forceps or a snare, depending on the size and shape of FB.
- Step 5: Gastric wall closure. In cases of unsatisfactory wound closure or severe injury, a nasogastric tube was inserted to mitigate the risk of delayed perforation or bleeding.

## Discussion

Antral-embedded FBs can trigger rapid submucosal growth, leading to inflammatory granulation tissue formation, which necessitates careful dissection and, in some cases, myotomy. The full-thickness defect of the gastric antrum is thicker than other regions, requiring meticulous closure even for defects <2 cm to ensure safety. Thus, the endoscopic removal of a gastric antral-embedded FB is both challenging and high-risk.

Several key factors must be considered during the procedure. Precise localization of the FB is paramount before making any incisions. CT imaging is recommended as the initial diagnostic step to detect embedded FBs and associated tissue changes, ruling out complications not suitable for endoscopic management [2]. Local mucosal bulging or edema induced by the FB can serve as excellent landmarks. Intraoperative EUS is strongly recommended for real-time localization [3–5].

A flexible combination of endoscopic accessories, such as retrieval baskets, nets, polypectomy snares, or transparent caps, may be essential in challenging cases [5]. Attaching a transparent cap to the endoscope tip improves visualization and prevents pharyngeal mucosal injury, especially when retrieving sharp FBs. For perforations <2 cm, metallic clips are the preferred closure method. Small superficial mucosal defects can be left open if there is no discernible damage to the muscle layer.

One of the six cases failed endoscopic extraction and turned to conservative treatment. During the procedure, the FB was “located” based on preoperative CT findings and a mucosal bulge with pus was observed endoscopically. However, after incision through the muscle layer, no FB was found in the submucosal layer or abdominal cavity. After 3 h of exploration, the incision was closed using hemostatic clips, and a nasogastric tube was inserted. The patient remained asymptomatic during the 1-year follow-up, with CT scans showing no signs of the FB. This case highlights the importance of utilizing real-time EUS decisively in cases of negative intraoperative findings for precise FB localization.

It is noteworthy that the endoscopic removal of embedded FBs is technically demanding [6]. The endoscopist must be familiar with gastric anatomy, proficient in safe dissection techniques and capable of closing full-thickness defects. As a disease-centered and individualized approach, endoscopic management of embedded FBs offers patients superior treatment options. With the continuous evolution of endoscopic techniques and instruments, we hold optimism about the future application of endoscopic resection in complex FB cases.

## Supplementary Material

goaf026_Supplementary_Data
